# Patterns of childhood-onset uveitis in a referral center in Turkey

**DOI:** 10.1007/s12348-011-0044-8

**Published:** 2011-10-16

**Authors:** Pinar Ç. Ozdal, Emine Sen, Alper Yazici, Faruk Ozturk

**Affiliations:** 1 Ulucanlar Eye Hospital, Ankara, Turkey; 2 Ulucanlar Eye Education and Research Hospital, Dikmen Cad. 434/12, 06450-Dikmen, Ankara, Turkey

**Keywords:** Epidemiology, Childhood, Pediatric, Uveitis, Complications, Prognosis

## Abstract

**Purpose:**

This study aimed to investigate the frequency and characteristics of childhood-onset uveitis and evaluate the rate and specific causes of visual loss in this population.

**Methods:**

The data of 121 patients (179 eyes) with uveitis starting before ≤16 years and followed up for at least 6 months were retrospectively evaluated. Age at onset, sex, laterality, associated systemic disease, laboratory data, therapeutic strategies, surgeries, final visual acuity, and causes leading to visual acuity ≤20/200 were analyzed.

**Results:**

Childhood-onset uveitis made up 9.6% of our uveitis patients. The mean age at onset was 11.7 years (1–16) and the mean follow-up period was 38.5 months (6–148). Forty-three patients (35.5%) were female and 78 were male (64.5%). The disease was bilateral in 58 (47.9%) and unilateral in 63 (52.1%) patients. Uveitis was mostly (59.5%) seen between 12 and 16 years of age. Pars planitis, observed in 29 (24%) patients, was the leading cause of childhood-onset uveitis. Uveitis was idiopathic in 20 (16.5%) of patients. The most frequently associated diseases were Behcet’s disease (BD) in 20 (16.5%), toxoplasmosis in 16 (13.2%), and juvenile idiopathic arthritis (JIA) in 8 (6.6%) patients. Anterior uveitis was observed in 38 (31.4%), intermediate uveitis in 31 (25.6%), posterior uveitis in 30 (24.8%), and panuveitis in 22 (18.2%) patients. The final visual acuity was ≤20/200 in 32/179 eyes (17.9%) of 27/121 patients (22.3%). The most often ocular complication leading to visual acuity ≤20/200 was optic atrophy and had been observed in 14 of 32 eyes (43.7%). Macular scar observed in five eyes (15.6%) was the second most often complication. Etiological distribution of 27 patients with visual acuity ≤20/200 was as follows: 12 had BD (44.4%), 5 had idiopathic uveitis (18.5%), 4 had pars planitis (14.8%), 3 had toxoplasmosis (11.1%), 2 had JIA (7.4%), and 1 had toxocara (3.7%).

**Conclusions:**

Although rare, childhood-onset uveitis has a blinding potential and causes visual loss in up to 22.3% of the patients. In endemic areas like Turkey, BD may be the most common uveitis-associated systemic disease and the leading cause of visual loss in childhood uveitis as adulthood.

## Introduction

Although uveitis is less common (5–10%) in children, the prognosis is nevertheless poorer [1]. Uveitis may be asymptomatic either because of the preverbal age of the children or the insidious nature of the disease [2–4]. Because the course of the disease is frequently chronic and the diagnosis is often delayed, children have a higher risk of complications [4]. The presence of severe ocular complications at the initial presentation is common [2]. The management of uveitis is associated with unique problems because of the patient’s immature immune system and developing skeletal and reproductive systems [3]. The incidence and associated systemic diseases show differences that may be due to geographic, genetic, demographic factors, and also to different referral patterns and definition of pediatric age range [3, 5]. Hence, pediatric uveitis is still a challenge for ophthalmologists. This study aimed to investigate the characteristics of childhood-onset uveitis and evaluate the rate and specific causes of visual loss in this particular population.

## Material and methods

We retrospectively evaluated the medical records of 1,265 uveitis patients presented at Uveitis and Behçet’s Disease Department of Ulucanlar Eye Hospital between January 1996 and May 2008 and retrieved 121 patients with uveitis starting before or at 16 years of age. All patients were immunocompetent. Patients with a follow-up period less than 6 months were excluded from the study. Age at onset, laterality and anatomic localization of uveitis, gender, follow-up period, associated systemic diseases, laboratory data, therapeutic strategies, performed surgical interventions, ocular complications, final visual acuity, and causes leading to visual acuity ≤20/200 were analyzed.

All patients underwent a clinical examination including visual acuity testing with Snellen chart, intraocular pressure measurement, biomicroscopy, and fundus examination through a dilated pupil by a 90D lens. Fundus fluorescein angiography, ultrasonography, and optical coherence tomography were performed when indicated. Routine laboratory evaluation consisted of a complete blood count with differential, biochemical analysis, urinalysis, and erythrocyte sedimentation rate. Antinuclear antibody, rheumatoid factor, C-reactive protein, human leukocyte antigen (HLA) typing, angiotensin-converting enzyme, pathergy test, skin tuberculin test, chest radiography, syphilis, toxoplasma, herpes, and lyme serology were done in selected patients. Patients were consulted with pediatricians or pediatric rheumatologists according to clinical findings.

Patients were classified as having anterior, intermediate, posterior, and panuveitis according to Standardization of Uveitis Nomenclature Working Group recommendations [6]. If the primary site of the inflammation was anterior chamber, then the uveitis was anterior, if the vitreous was the primary site, then the uveitis was intermediate, and if retina or choroid was primarily involved, then the uveitis was posterior. Uveitis involving all of these anatomical parts was diagnosed as panuveitis. Pars planitis was considered as a specific uveitis entity and this term was used only for intermediate uveitis where there is a snowbank or snowball formation occurring in the absence of an associated infection or systemic disease (that is, “idiopathic”). If there is an associated infection or systemic disease, then the term intermediate uveitis was used. The diagnosis of ocular toxoplasmosis and toxocariasis was based on typical clinical features and confirmed by positive serology. Patients having uveitis without an associated systemic disease, laboratory abnormality, or well-established specific uveitis entity such as Fuchs' uveitis, pars planitis, and herpetic uveitis were considered to be idiopathic. Uveitis with sudden onset lasting ≤3 months was considered as acute, and uveitis lasting longer than 3 months and relapsing in <3 months after discontinuation of treatment was considered as chronic uveitis. The diagnosis of Behçet’s disease was made according to the criteria of the International Study Group for Behçet’s Disease [7].

Treatment modalities applied to treat either uveitis or its complications were classified as topical, systemic, periocular, intraocular, and surgical. Medical treatment was instituted regarding the etiology and the severity of uveitis. Surgical treatment included cataract extraction, pars plana vitrectomy, pars plana lensectomy, glaucoma surgery, chelation with ethyldiamietetraacetic acid (EDTA), and peripheral cryotherapy.

## Results

Cases of childhood-onset uveitis made up 9.6% (121/1265) of our uveitis patients. Forty-five patients (37.2%) were female and 76 (62.8%) were male. Mean age at onset of uveitis was 11.7 ± 3.6 years (range 1–16). Of 121 patients, 72 (59.5%) were between 12 and 16, 41 (33.9%) were between 6 and 11, and 8 (6.6%) were between 0 and 5 years old. Thus, the uveitis was mostly seen between 12 and 16 years of age. The mean follow-up period was 38.5 ± 3.2 months (6–148).

Uveitis was infectious in 26 patients (21.5%) and noninfectious in 95 (78.5%) patients. Pars planitis was found to be the most common cause of uveitis (29 patients, 24%) in pediatric cases. Uveitis was idiopathic in 20 (16.5%) patients and BD was the most frequently associated systemic disease (20 patients, 16.5%). Toxoplasmosis observed in 16 patients (13.2%) was the most common cause of posterior and infectious uveitis. Other less common causes of uveitis were JIA (eight patients, 6.6%), herpes infection (five patients, 4.1%), and ankylosing spondilitis (five patients, 4.1%). Table 1 shows etiologies of uveitis and their distribution by gender.

**Table 1:** Etiology of uveitis by gender and laterality

Diagnosis	Female	Male	Unilateral	Bilateral	No. of patients (%)	No. of eyes (%)
Pars planitis	8	21	4	25	29 (23.9)	54 (30.2)
Idiopathic uveitis	8	12	12	8	20 (16.5)	28 (15.6)
Behcet’s disease	8	12	8	12	20 (16.5)	32 (17.8)
Toxoplasmosis	10	6	15	1	16 (13.2)	17 (9.5)
JIA	4	4	4	4	8 (6.6)	12 (6.7)
Herpetic uveitis	1	4	5	–	5 (4.1)	5 (2.8)
Ankylosing spondilitis	2	3	2	3	5 (4.1)	8 (4.5)
Toxocara		4	4	–	4 (3.3)	4 (2.2)
Fuchs’ uveitis		3	3	–	3 (2.5)	3 (1.7)
Traumatic iritis		3	3	–	3 (2.5)	3 (1.7)
FMF		2	–	2	2 (1.6)	4 (2.2)
Multifocal choroiditis	1	1	2	–	2 (1.6)	2 (1.1)
Sarcoidosis	1		–	1	1 (0.8)	2 (1.1)
Brucella		1	1	–	1 (0.8)	1 (0.5)
VKH	1		–	1	1(0.8)	2 (1.1)
Diabetic iritis	1		–	1	1 (0.8)	2 (1.1)
Total	45	76	63	58	121 (100)	179 (100)

*JIA* juvenile idiopathic arthritis, *FMF* Familial Mediterranean Fever, *VKH* Vogt–Koyanagi–Harada disease

Anterior uveitis was observed in 38 (31.4%), intermediate uveitis in 31 (25.6%), posterior uveitis in 30 (24.8%), and panuveitis in 22 (18.2%) patients. While idiopathic uveitis and JIA were the most common causes of anterior uveitis, toxoplasmosis was the leading cause of posterior uveitis. Panuveitis was mostly observed in BD-associated uveitis. Table 2 shows anatomic localization of uveitis according to etiology.

**Table 2:** Anatomic localization of uveitis according to etiology

Diagnosis	Anterior	Intermediate	Posterior	Panuveitis
Pars planitis		27		2
Idiopathic uveitis	10	1	4	5
Behcet’s disease	4	2	4	10
Toxoplasmosis			14	2
JIA	6			2
Herpetic uveitis	5			
Ankylosing spondilitis	5			
Toxocara			4	
Fuchs’ uveitis	3			
Traumatic iritis	3			
FMF	1		1	
Multifocal choroiditis			2	
VKH				1
Brucella			1	
Diabetic iritis	1			
Sarcoidosis		1		
	38 (31.4%)	31 (25.6%)	30 (24.8%)	22 (18.2%)

*JIA* juvenile idiopathic arthritis, *FMF* Familial Mediterranean Fever, *VKH* Vogt–Koyanagi–Harada disease

The disease was bilateral in 58/121 (47.9%) patients resulting in 179 affected eyes. Herpetic uveitis (5/5, 100%), ocular toxoplasmosis (15/16, 93.7%), and idiopathic uveitis (12/20, 60%) had a tendency to be unilateral, whereas ocular BD (12/20, 60%) and pars planitis (25/29, 86.2%) had a tendency to be bilateral. Uveitis was acute in 39 (32.2%) and chronic in 82 (67.8%) patients.

Laboratory evaluation showed HLA B51 positivity in 18 (14.9%) patients in whom nine (50%) had BD-associated uveitis. Thus, the rate of HLA B51 positivity was 45% (9/20 patients) in BD patients. HLA B27 was positive in three patients with ankylosing spondylitis and HLA DR15 was positive in five patients with pars planitis. Toxoplasmosis IgG was positive and IgM negative in all patients with ocular toxoplasmosis. Herpes simplex virus type I IgM was positive in one and IgG in two patients with herpetic uveitis. Antinuclear antibody was positive in two patients with JIA-associated uveitis and one patient with pars planitis. Angiotensin-converting enzyme level was high in four patients in whom two had the diagnosis of pars planitis, one sarcoidosis, and one idiopathic uveitis.

Complications secondary to uveitis were cataract (38 eyes, 21.2%), glaucoma (32 eyes, 17.8%), cystoid macular edema (CME) (17 eyes, 9.5%), vitreous condensation (15 eyes, 8.4%), optic atrophy (14 eyes, 7.8%), band keratopathy (13 eyes, 7.3%), posterior capsule opacification, retinal detachment and macular scar (5 eyes, 2.8% for each), pupillary occlusion (3 eyes, 1.8%), macular hole, and neovascularization of the disc (1 eye, 0.6% for each) (Table 3).

**Table 3:** Complications secondary to childhood uveitis

	*N* (eyes)	%
Cataract	38	21.2
Glaucoma	32	17.8
Cystoid macular edema	17	9.5
Vitreus condensation	15	8.4
Optic atrophy	14	7.8
Band keratopathy	13	7.3
Posterior capsule opacification	5	2.8
Retinal detachment	5	2.8
Macular scar	5	2.8
Pupillary occlusion	3	1.8
Macular hole	1	0.6
Disc neovascularization	1	0.6

Topical treatment was needed in 73 patients (60.3%). Systemic treatment included immunosuppressive and/or immunomodulator therapy in 32 patients [14 with BD, 7 JIA, 8 pars planitis, 1 ankylosing spondylitis, 1 Familial Mediterranean Fever (FMF), and 1 Vogt–Koyanagi–Harada disease (VKH)], antibiotic therapy in 10 patients (9 with ocular toxoplasmosis and 1 brucellosis), and antiherpes therapy in 3 patients. Systemic corticosteroids were used in 23 patients in combination with either immunosuppressive agents or antibiotics.

Posterior subtenon injections of triamcinolone acetonide in 50 (27.9%), intravitreal injections of triamcinolone acetonide in 9 (5%), and intravitreal injection of bevacizumab in 3 (1.7%) eyes were performed. Laser photocoagulation (four eyes, 2.2%) and Nd-YAG capsulotomy (ten eyes, 5.6%) were applied in required cases.

Performed surgeries were as follows: cataract extraction with or without intraocular lens implantation (22 eyes, 12.3%), pars plana vitrectomy (14 eyes, 7.8%), trabeculectomy (6 eyes, 3.3%), EDTA scrub (3 eyes, 1.7%), retinal detachment surgery (2 eyes, 1.1%), pars plana vitrectomy and lensectomy (1 eye, 0.6%), enucleation (1 eye, 0.6%), and intraocular lens extraction (1 eye, 0.6%). Some of these surgeries were performed in our clinic during the follow-up period, but some were already performed at the time of presentation. We did not implant intraocular lens to children with JIA and a child with VKH having chronic inflammation with pupillary occlusion. Intraocular lens was implanted to patients with BD, pars planitis, Fuchs’ uveitis, FMF, and idiopathic uveitis in order to be quiet for at least 3 months. A painful eye with phthisis bulbi due to BD-associated uveitis and neovascular glaucoma was enucleated.

Visual acuities at first presentation to our Uveitis and Behçet’s Disease Department were ≥20/32 in 97 eyes (54.2%), between 20/100 and 20/40 in 45 eyes (25.1%), and ≤20/200 in 37 eyes (20.7%). The number of patients having visual acuity ≤20/200 in at least one eye was 29 (24%).

Visual acuities at last visit were ≥20/32 in 125 eyes (69.8%), between 20/100 and 20/40 in 22 eyes (12.3%), and ≤20/200 in 32 eyes (17.9%). The number of patients having final visual acuity ≤20/200 in at least one eye was 27 (22.3%). Of these 27 patients, 10 had BD (37%), 5 had idiopathic panuveitis (18.5%), 5 had pars planitis (18.5%), 4 had ocular toxoplasmosis (14.8%), 2 had JIA-associated panuveitis (7.4%), and 1 had toxocara (3.7%). Thus, 22 of 27 patients (81.5%) had either panuveitis or posterior uveitis. Optic atrophy was the main cause of visual loss and has been observed in 14 eyes (43.7%). Ten of 14 eyes with optic atrophy had BD-related posterior or panuveitis. Others were toxocara (one eye), VKH (one eye), brucella (one eye), and idiopathic panuveitis (one eye). Macular scar was the second most frequent complication and was observed in five eyes (15.6%). Band keratopathy, CME, macular hemorrhage, severe vitritis, retinal detachment, macular hole, retinal fibrosis, and enucleated eye were other causes leading to visual loss.

## Discussion

Childhood uveitis is a potentially blinding disease characterized by a chronic course and a high (76%) complication rate [8]. Its association with systemic diseases is different than adulthood and varies from one study to another (Table 4). Race and ethnicity were reported to associate with different patterns of uveitis, disease presentation, and progression [9]. In most of the previous series [2, 8–14], the majority of cases (24.2–60%) have been reported to be idiopathic which is not consistent with our results. Idiopathic uveitis was the second most common cause of childhood uveitis in our series and has been identified in 16.5% of patients.

**Table 4:** Causes of pediatric uveitis (a summary of previously published series)

	Perkins [10]; (%)	Kanski [15]; (%)	Giles [2]; (%)	Tugal-Tutkun [3]; (%)	Soylu [13]; (%)	Kadayıfçılar [12]; (%)	Current Study
Pars planitis	–	–	–	15.3	8.9	11.87	24
Idiopathic	43.3	5.8	60	21.5	34.4	24.2	16.5
Behcet’s disease	–	0.5	–	0.7	11.1	10.96	16.5
Toxoplasmosis	20.6	–	–	7.7	25.6	21	13.2
JIA	5.3	76.7	20	41.5	3.3	13.24	6.6
Herpetic uveitis	3.3	–	3.4	–	3.3	2.74	4.1
Toxocara	10	–	–	3.1	1.1	3.2	3.3
Ankylosing spondylitis	0.6	12.7	1.3	0.7	–	1.83	4.1
Fuch’s uveitis	2.6	–	3.9	0.7	5.6	4.11	2.5
Brucella							0.8
Sarcoidosis	2.6	0.8	3.9	2.3	–	0.91	0.8
FMF							1.6
VKH	0.6	0.2	–	1.5	–	0.46	0.8
Tuberculosis	3.3	–	1.7	–	–	1.37	–

*JIA* juvenile idiopathic arthritis, *FMF* Familial Mediterranean Fever, *VKH* Vogt–Koyanagi–Harada disease

Juvenile idiopathic arthritis is the leading associated systemic disease in most of the previous studies on childhood uveitis [2, 3, 5, 8, 9, 15–17]. Furthermore, in the study of Tugal-Tutkun et al. [3] and Kanski et al. [15], JIA-associated uveitis makes up the primary cause of childhood uveitis. Foster stated that in many tertiary referral centers, up to 80% of anterior uveitis cases seen in children are as a consequence of JIA [18]. JIA-associated uveitis which has been observed in 6.6% of our patients was the fifth most frequent cause of childhood-onset uveitis. This relatively low frequency might be explained by genetic and demographic factors playing role in the occurrence of JIA. Previous studies from Turkey reported similar results regarding the frequency of JIA-associated uveitis [12, 13].

Toxoplasmosis and pars planitis are reported to be other frequent causes of childhood uveitis by several previous studies [1, 3, 9, 11–13, 17]. Pars planitis is described as an intermediate uveitis where there is a snowbank or snowball formation occurring in the absence of an associated infection or systemic disease [6]. Although idiopathic, this specific uveitic entity was considered separately and not included in idiopathic uveitis group. Pars planitis was the leading cause of uveitis in our series with a rate of 24%. Ocular toxoplasmosis has been observed in 13.2% and made up the main cause of infectious uveitis in accordance with previous studies. Other causes of infectious uveitis were herpes, toxocara, and brucella infections. Infectious uveitis made up 21.5% of our cases and this rate was slightly lower than that of previous two studies from Turkey reporting infectious causes in 27% and 30% of pediatric cases [12, 13]. Infectious causes were identified in 5–24.3% of patients in other reports [4, 8, 14, 19].

Behçet’s disease-associated uveitis has been observed in 0.5–2.2% of patients with childhood-onset uveitis in nonendemic areas [3, 15, 17]. Behçet’s disease which has been observed in 16.5% of our patients was the second most common cause of uveitis with idiopathic uveitis which has exactly the same rate and number of patients. Additionally, BD was the most commonly associated systemic disease. This rate was even higher than that of Soylu et al. (11.1%) which is the highest previously reported rate of ocular BD in children [13]. BD was the fifth most common cause of uveitic entity with a rate of 11% in another study from Turkey by Kadayıfçılar et al. [12].

Less common causes of childhood uveitis in our series were ankylosing spondilitis, Fuchs’ uveitis, trauma, multifocal choroiditis, FMF, VKH, sarcoidosis, and diabetes. A higher prevalence of BD among FMF patients has been described compared to the general population [20]. Furthermore, a recent study of JIA patients in Turkey revealed that 21 of 634 patients also had FMF [21]. In the light of these data, FMF-associated uveitis is not an unexpected condition, although not reported before. None of our patients with FMF fulfilled the criteria of the International Study Group for Behçet’s Disease. It is thought that these patients may develop BD later in their life.

As its causes, forms of uveitis in children differ from those in adults. Cunningham reviewed nine large studies coming mostly from tertiary care facilities and found that posterior uveitis was slightly more common than anterior uveitis in children and accounted 40% to 50% of cases versus 30% to 40%. Whereas in adults anterior uveitis was more common (50%) than posterior uveitis (20%) [22]. However, a literature review by Holland and Stiehm suggested that in population-based studies, the majority of uveitis in children is restricted in anterior segment [23]. Consistently with this review and studies of Rosenberg et al. (30.4%) [4], Smith et al. (44.6%), and Kump et al. (57%) [19], anterior uveitis was found to be more common (31.4%) in our study. We should keep in mind that the presentation of uveitis may change over time. Uveitis may start at the anterior part of the eye and involve the vitreous and the retina in course of time. Similarly, uveitis initially called idiopathic may fit a particular category later. Thus, the investigations should be repeated from time to time. Childhood uveitis has been reported to be mostly bilateral [9, 14]. On the contrary, unilateral involvement was slightly more (52.1%) in our patients. We know that uveitis may start at one eye and tend to be bilateral during the course of the disease. It is certain that some of these patients will have bilateral involvement later.

Childhood uveitis was described as a potentially blinding disease associated with visual loss in 19%, complication(s) in 76%, and surgical intervention in 28% of patients [8]. Rosenberg et al. [4] reported any kind of complications in 94.6% of patients including macular complications (70.9%), posterior synechia (54.7%), cataract (52%), optic nerve complications (36.5%), band keratopathy (35.1%), and glaucoma (33.1%). Optic atrophy (43.7%), cataract (21.2%), glaucoma (17.8%), and cystoid macular edema (9.5%) were the most common complications related to uveitis in our pediatric patients. The rate of visual loss has been reported to be 17.5% in another study [3]. In the largest series of childhood uveitis, the proportion of patients with vision ≤20/200 was 9.2 at baseline, 6.5% at 1 year, 15.1% at 5 years, and 7.7% at 10 years. Of our patients, 22.3% had at least one legally blind eye (a visual acuity ≤20/200) at final examination. Our higher rate for visual loss might be explained with our higher incidence for BD. Behçet’s disease-associated uveitis made up 37% of our patients with blind eye. Among 20 patients with BD, ten (50%) had at least one legally blind eye at final visit. A significant number of these patients were referrals with severe ocular involvement at presentation and their systemic therapy had been delayed. Most of these patients (14/20) presented with panuveitis or posterior uveitis and relating complications leading to visual loss. As reported by Tugal-Tutkun and Urgancioglu, this group of patients needs a prompt immunosuppressive therapy [24]. Blindness due to childhood BD has been reported in 16.6% of patients in the abovementioned study from Turkey [24] and in none of the patients in a study from Germany [25]. It is known that besides differences in geographic and ethnic origin of patients, individual factors may play an important role in variable expression and severity of the disease [20]. Posterior and panuveitis in children are considered poor prognostic markers [9]. Consistently, most of our patients (81.5%) with legally blind eye had either panuveitis or posterior uveitis. The fact that 24% of patients already had a visual loss at their first presentation to the clinic might be another explanation for worse visual prognosis.

## Conclusion

Childhood-onset uveitis has a relatively severe course and led to visual loss in up to 22.3% of our patients. Difficulties in diagnosing and treating the patients, high risk of complications, and ambliopia are all challenges in this particular population. Etiological distribution of uveitis is an additional factor affecting the visual prognosis. Differently from studies of Western populations, Behçet’s disease was the most commonly associated systemic disease in childhood-onset uveitis in our population. This finding is not surprising with the fact that Turkey has the highest prevalence rate of the disease in the world. Our high rate of BD-associated uveitis (16.5%) is the most important factor explaining the high rate of permanent visual loss. Causes and associated systemic diseases and also the course of uveitis are closely related to differences in ethnic and geographic origins of patients. To be aware of the causes leading to uveitis in the pediatric population of a specific community is very helpful for a prompt diagnosis and treatment and also the prevention of severe complications.
